# Anti-GPC3 antibody-modified sorafenib-loaded nanoparticles significantly inhibited HepG2 hepatocellular carcinoma

**DOI:** 10.1080/10717544.2018.1477859

**Published:** 2018-06-19

**Authors:** Xiaolong Tang, Longzhou Chen, Amin Li, Shiyu Cai, Yinci Zhang, Xueke Liu, Zhenyou Jiang, Xinkuang Liu, Yong Liang, Dong Ma

**Affiliations:** a Medical College, Anhui University of Science and Technology, Huainan, China;; b Department of Galactophore, Huai'an Maternity and Child Healthcare Hospital Affiliated to Yangzhou University Medical Academy, Huaian, China;; c Department of Microbiology and Immunology, Jinan University, Guangzhou, China;; d Huai’an Hospital Affiliated of Xuzhou Medical College and Huai’an Second Hospital, Huai’an, China;; e Department of Biomedical Engineering, Key Laboratory of Biomaterials of Guangdong Higher Education Institutes, Jinan University, Guangzhou, China

**Keywords:** Hepatocellular carcinoma, sorafenib, anti-GPC3 antibody, targeted delivery, nanoparticles

## Abstract

Sorafenib (SFB) has improved the treatment of hepatocellular carcinoma (HCC) and has fewer severe side effects than other agents used for that purpose. However, due to a lack of tumor-specific targeting, the concentration of the drug in tumor tissue cannot be permanently maintained at a level that inhibits tumor growth. To overcome this problem, we developed a novel SFB-loaded polymer nanoparticle (NP). The NP (a TPGS-*b*-PCL copolymer that was synthesized from ε-caprolactone and d-α-tocopheryl polyethylene glycol 1000 succinate (TPGS) via ring-opening polymerization) contains Pluronic P123 and SFB, and its surface is modified with anti-GPC3 antibody to produce the polymer nanoparticle (NP-SFB-Ab). The Ab-conjugated NPs had higher cellular uptake by HepG2 cells than did non-antibody-conjugated SPD-containing nanoparticles (NP-SFB). The NP-SFB-Ab also displayed better stability characteristics, released higher levels of SFB into cell culture medium, and was more cytotoxic to tumor cells than was non-targeted NP-SFB and free SFB. The NP-SFB-Ab downregulated expression of the anti-apoptosis molecule MCL-1, which led to polymerization of Bax and Bak in mitochondrial cytosol. The NP-SFB-AB also promoted the mitochondrial release of cytochrome C, resulting in cellular apoptosis. Moreover, the NP-SFB-Ab significantly inhibited the growth of HepG2 xenograft tumors in nude mice without producing obvious side effects. These findings suggest that NP-SFB-Ab is a promising new method for achieving targeted therapy of HCC.

## 1. Introduction

Hepatocellular carcinoma (HCC) is the third most deadly type of cancer worldwide (Chiang & Villanueva, 2017; Nakamura et al., 2018). However, there has not been a major advance in the systematic treatment of liver cancer for many years. During the past 10 years, numerous experimental drugs for treating HCC have been studied in the laboratory and clinic, but they eventually failed due to their toxicity to normal tissues, leaving sorafenib (SFB) the only standard first-line pharmacologic agent for treating HCC. Currently, there is no second-line treatment for advanced HCC. SFB is a multi-kinase inhibitor with both anti-tumor cell and anti-tumor vascular effects, and it has been shown to improve the survival times of HCC patients (Parikh et al., 2017; Pinter et al., 2017; Won et al., 2017). However, some cases of HCC are refractory to SFB (Won et al., 2017; Zhu et al., 2017), so it is important to develop new methods for overcoming this problem. Polymeric nanocarriers increase the solubility of hydrophobic drugs, prolong their half-life, enhance the targeting of drugs to tumors, and can be used to overcome multidrug resistance by enhancing drug permeation and retention in tumor cells (Zhou et al., 2017). Due to its unique properties, polycaprolactone (PCL) is one of the most widely used polymers. PCL is biodegradable, miscible with other polymers, nontoxic, and easy to crystallize (Li et al., 2017; Totaro et al., 2017). Copolymers of TPGS-b-PCL such as PCL plus d-α-tocopheryl polyethylene glycol 1000 succinate (TPGS) have been widely used to deliver antitumor drugs such as genistein and paclitaxel (Tang et al., 2014; Parikh et al., 2017). TPGS is a water-soluble form of vitamin E that can prolong drug circulation times, inhibit P-glycoprotein activity, and be used in combination with other anticancer drugs (Lian et al., 2017; Tang et al., 2017). While copolymerization of PCL with TPGS makes PCL more hemocompatible, PCL still retains its high degree of hydrophobicity and slow degradation and drug release properties, which limit its applications. To solve these problems, we added Pluronic^®^ P123 to PCL. Pluronic compounds are widely used in the biomedical field for purposes ranging from drug delivery to medical imaging (Guha et al., 2016; Forget et al., 2017; Liu et al., 2017). Pluronic P123 can be added to PCL as a nucleating agent to enhance drug release. Moreover, Pluronic compounds have been reported to interact with multidrug resistant tumors and increase their sensitivity to anticancer drugs (Sharma et al., 2015). Pluronic compounds are transported into cells upon their insertion into the cell membrane, and they subsequently affect cellular functions such as mitochondrial respiration, ATP synthesis, and drug transport systems. Therefore, the use of TPGS-b-PCL in conjunction with a Pluronic compound can encapsulate a drug, control its release, prolong its circulation time, and possibly enhance its anti-tumor effect. For example, Mei et al. (2009) used paclitaxel-loaded PCL/Pluronic F68 nanoparticles (NPs) to overcome multidrug resistance in breast cancer.

Finally, the binding of an active target ligand to a nanocarrier is a method that is widely used to achieve specific cell recognition and enhance the targeting of therapeutic agents to cancer cells (Zhu et al., 2001; Kim et al., 2011; Avanesov et al., 2012). Glypican-3 (GPC3), a member of the glypican family, is anchored by glycosylphosphatidylinositol (GPI) (Zhu et al., 2001; Kim et al., 2011; Avanesov et al., 2012), and highly expressed in most liver cancers, but it is absent or expressed at very low levels in normal adult tissues (Kim et al., 2011; Avanesov et al., 2012). Numerous anti-GPC3 monoclonal antibodies target GPC3 molecules that recognize the membrane architecture of HCC cells, resulting in antibody-mediated endocytosis (Zhu et al., 2001). Conjugation of an anti-GPC3 antibody to a nanocarrier should enhance the targeted delivery of drugs to cancers cells. We designed a novel SFB-loaded polymer NP to enhance the treatment of liver cancer. This polymer NP, designated as NP-SFB-Ab, was prepared from SFB, TPGS-b-PCL, and Pluronic P123-Mal by nanoprecipitation followed by conjugation to an anti-GPC3 antibody. The following physico-chemical properties of the resulting NP-SFB-Ab particles were characterized: particle size, zeta potential, surface properties, drug loading, and drug entrapment efficiency. We also examined the ability of NP-SFB-Ab to inhibit HepG2 cell proliferation and its mechanism of action.

## 2. Materials and methods

### 2.1. Materials

d-α-Tocopheryl polyethylene glycol 1000 succinate (TPGS) and ε-caprolactone (ε-CL) were purchased from Sigma-Aldrich (St. Louis, MO). Hydrazine hydrate (50%), Pluronic P123 (*M*w = 5800), N-succinimidyl 3-maleimidopropionate (SMMP), stannous octoate (Sn(Oct)2), 4-toluene sulfochloride, Traut's reagent (2-iminothiolane), phthalimide potassium salt, and trimethylamine were purchased from Alfa Aesar (Ward Hill, MA). Human hepatic cancer (HepG2) cells and human lactoferrin (HLF) cells were obtained from the American Type Culture Collection (Manassas, VA). The culture medium used in these experiments was Dulbecco's modified Eagle's medium (DMEM) or 10% fetal bovine serum (FBS). l-Glutamine and penicillin-streptomycin were purchased from Gibco (Gaithersburg, MD), and PCR primers were purchased from Shanghai Biotech (Shanghai, China). SFB was purchased from Celik Chemical Company (Houston, TX). Anti-GPC3 mAb (Clone 9C2) was purchased from Biomosaics Inc. (Burlington, VT); primary antibodies for Mcl-1, Bax, B-Raf, C-Raf, pMEK_1/2_, pERK, cleaved caspase-8, cleaved caspase-3, X-linked inhibitor of apoptosis protein (XIAP), and cytochrome c were purchased from Santa Cruz Biotechnology (Santa Cruz, CA). Primary antibodies for Bad and XIAP were purchased from BD Biosciences (Bedford, MA); anti-VEGF, anti-PARP, and anti-Bak antibodies were obtained from ALEXIS Corporation (San Diego, CA). Cellular FLICE (FADD-like IL-1β-converting enzyme)-inhibitor protein (c-FLIP) was purchased from Cell Signaling Technology, Inc. (Danvers, MA). Propidium iodide (PI) was obtained from Sigma Chemical Co. (St. Louis, MO); other chemicals are commercially available.

### 2.2. Synthesis of the TPGS-b-PCL polymer and pluronic P123-Mal

**Figure 1.**
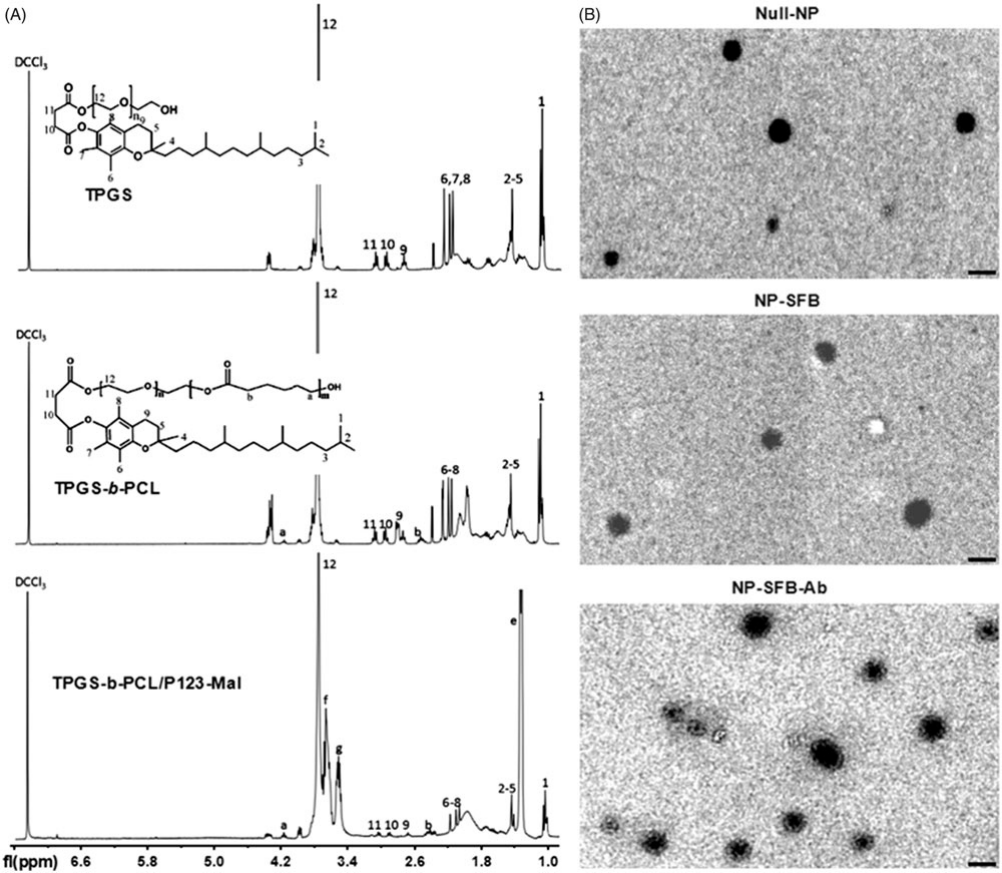
^1^H NMR spectra and TEM images of synthetic copolymers. (A) ^1^H NMR spectra of TPGS, TPGS-*b*-PCL and TPGS-*b*-PCL/P123-Mal copolymer: 4.08 ppm and 2.37 ppm were the peaks for –OCCH2– and –CH2OOC– on PCL, respectively; the peak at 3.66 ppm was due to the methylene protons of TPGS. 7.28 ppm was the peak of CDCl_3_ solvent; 3.57, 3.42, and 1.15 ppm were the characteristic peaks of P123; 3.66 ppm is the characteristic peak of TPGS, proving the successful combination of P123-Mal and TPGS-b-PCL. (B) Transmission electron micrographs (TEM) of Null-NP, NP-SFB, and NP-SFB-Ab. Bar: 100 nm.

The reaction scheme used to synthesize the TPGS-*b*-PCL copolymer is shown in Figure 1(S)(A). In brief, 2 g of ε-CL and 2 g of water-soluble vitamin E were added to a flask at 40 °C and vacuum dried overnight. Next, 1–2 drops of stannous octoate was added to the flask, which was then sealed. Next, the flask was purged with nitrogen at room temperature, vacuum pumped several times for 10 minutes each time, and repeatedly ventilated and aspirated. The flask was placed in an 80 °C oil bath to melt the TPGS, after which, it was placed into an ice bath to solidify the TPGS and vacuum pumped three times. The reaction system was heated to 150 °C and allowed to react for 12 h. The reaction product was purified by dissolution in methylene chloride and precipitated with petroleum ether. TPGS-*b*-PCL was obtained by rotary evaporation, with a yield of 71%. The chemical structure of the synthesized TPGS-*b*-PCL copolymer was verified by proton nuclear magnetic resonance (^1^H NMR) spectroscopy (Bruker-300, Bruker Avance Co., Billerica, MA).

Pluronic P123-Mal was synthesized as described in Figure 1(S)(B). Pluronic P123 (4.6 g) was dissolved in 20 mL of CH_2_Cl_2_, followed by the addition of 4-toluene sulfochloride (4.6 g) and trimethylamine (2.5 mL). This mixture reacted at room temperature for 12 h, after which a purification step yielded p-toluenesulfonyl Pluronic P123 (Pluronic P123-OTs). The Pluronic P123-OTs (3.5 g) were reacted with phthalimide potassium salt in DMF at 130 °C for 5 h to obtain Pluronic P123-PI. Anhydrous ethanol (15 mL) and hydrazine hydrate (50%, 1 mL) were added to the Pluronic P123-PI, and the mixture was refluxed for 12 h to obtain Pluronic P123-NH_2_. Finally, the Pluronic P123-NH_2_ was reacted with 1.2 N N-succinimidyl 3-maleimidopropionate (SMMP) at room temperature for 2 h to obtain Pluronic P123-Mal. The structure of Pluronic P123-Mal was verified by ^1^H NMR.

### 2.3. Formation of SFB-loaded polymeric nanoparticles and antibody conjugation

The polymeric nanoparticle NP-SFB was prepared by a modified nanoprecipitation method which employed an acetone–water system. In brief, a pre-weighed amount of SFB powder and 100 mg of TPGS-*b*-PCL and Pluronic P123-Mal polymers (weight ratio of TPGS-*b*-PCL:Pluronic P123-Mal = 3:1) were dissolved in 8 mL of acetone by vortex mixing plus sonication. This solution was added dropwise to 100 mL of an aqueous solution of 0.03% TPGS with stirring. The resulting suspension was stirred uncovered overnight to completely remove any acetone. The NP suspension was centrifuged at 25,000 rpm for 1 h and washed three times to remove the emulsifier TPGS and any nonencapsulated drug. Finally, the dispersed solution was lyophilized for two days and stored for further use.

The antibody was reacted with Traut’s reagent (2-iminothiolane HCl, 2-IT) to modify its thiol groups to Ab-SH. Next, NP-SFB-Ab was prepared by mixing NP-SFB and Ab-SH at room temperature during centrifugation at 300 rpm for 2 h (Figure 1S). Fluorescent coumarin 6-loaded micelles were prepared similarly. The lyophilized NPs were re-dispersed in PBS before use.

### 2.4. Characterization of the SFB-loaded polymeric nanoparticles

#### 2.4.1. Size, zeta potential, and morphology

The size and zeta potential of NP-SFB-Ab and NP-SFB in PBS were determined using a Malvern Mastersizer 2000 (Zetasizer Nano ZS90, Malvern Instruments, Malvern, UK). The tests were performed three times, and the mean values were calculated. The morphologic characteristics of the micelles were viewed under a transmission electron microscope (TEM, Tecnai G2 F30, FEI Company, Hillsboro, OR). The mass of Ab on the NP surface was quantified using the bicinchoninic acid (BCA) assay. Briefly, a BCA working solution was prepared by mixing 50 volumes of reagent A with one volume of reagent B. A standard curve for the Ab was established after using native Ab to produce a series of Ab concentrations (0.125–2 mg/mL). The assays were performed by adding 10 μL of Ab or NP-Ab solution into each well of a 96-well plate, followed by the addition of 200 μL of BCA working solution. The plate was then incubated at 60 °C for 30 min. After the solution was cooled to room temperature, the absorbance of each well at 562 nm was determined with a UV-Visible spectrometer.

#### 2.4.2. Drug loading and drug encapsulation efficiency

The drug loading content (LC) and drug encapsulation efficiency (EE) of the SFB-loaded NPs were determined as described (Lin et al., 2016). SFB-loaded NPs were centrifuged at 14,000 rpm for 30 min, and the supernatant fractions were collected and analyzed by UV spectroscopy (Multiskan Spectrometer, Thermo Scientific, Rockford, IL) at 270 nm. The vehicle background absorption was subtracted by measuring the absorption of the supernatants of NPs prepared without SFB under the same conditions. All measurements were performed in triplicate. The LC and EE values of the SFB-loaded NPs were calculated using the following formulas:

LC%=(weight of SFB in the NPs)/(weight of the NPs)×100%

EE (%)=(weight of SFB in the NPs)/(weight of feeding SFB)×100%

#### 2.4.3. *In vitro* stability and drug release study

The stability of NP-SFB-Ab and NP-SFB in cell-culture medium containing 10% FBS was determined over 14 d by dynamic light scattering (DLS) (Liu et al., 2017). The drug release rates of the NPs under similar conditions were studied with the dialysis method. Briefly, a 10 mL volume of dissolved NPs (1 mg/mL) was placed into a dialysis bag (MWCO: 3 kDa) that was then incubated in a tube containing 30 mL of cell culture medium that contained 10% serum (pH 7.4). The tube was placed in an orbital shaker water bath and vibrated at 150 rpm at 37 °C. The solution outside the dialysis bag was sampled at defined periods, and the concentrations of SFB were measured by UV spectroscopy at 270 nm.

### 2.5. Cellular uptake of coumarin 6-loaded polymeric nanoparticles

The cellular uptake of coumarin-6 loaded NPs was analyzed by confocal laser scanning microscopy (CLSM). HepG2 cells were seeded into 12-well culture plates at a density of 1 × 10^4^ cells per well and cultured overnight in DMEM containing 10% FBS. On the following day, the cells were washed with PBS and incubated with coumarin 6-loaded NP-Ab or coumarin 6-NPs with coumarin 6 dose of 2 μg/mL in medium containing 10% serum for 4 h at 37 °C. Next, the cell nuclei were stained with DAPI, after which, the cells were washed, fixed, and detected by CLSM (Olympus Fluoview FV-1000, Tokyo, Japan).

### 2.6. Biocompatibility *in vitro*

As medicines contained in NPs are transported via the circulatory system, we measured the hemolytic effects of free SFB, NP-SFB, and NP-SFB-AB to determine their hemocompatibility (Sharma et al., 2015). Briefly, erythrocytes were isolated from the peripheral blood of healthy mice. Next, 0.4 mL aliquots of PBS containing various concentrations (0–10 μM) of free SFB or SFB-loaded nanospheres were mixed with 0.6 mL of a 0.2% mouse erythrocyte (v/v) suspension and incubated for 48 hours at 37 °C, after which, the suspensions were centrifuged at 700 rpm for five minutes. The rate of drug-induced hemolysis was calculated by measuring the optical density (OD) of the supernatant at 545 nm with a spectrophotometer (Model 1700; Shimadzu Corp., Kyoto, Japan). The hemolysis rate (%) was calculated as follows:

$$\text{Hemolysis rate }\left(\%\right)=\left({\text{OD}}_{\text{sample}}-{\text{OD}}_{\text{negative control}}\right)/\left({\text{OD}}_{\text{positive control}}-{\text{OD}}_{\text{negative control}}\right)\times \text{1}00\%$$

EA.hy926 vascular endothelial cells were cultured in 96-well plates (1 × 10^4^ cells/well) containing DMEM (10% FBS) in a humidified atmosphere of 5% CO_2_ at 37 °C. After 24 hours of culture, the growth medium was replaced with 300 μL of DMEM medium containing the required amount of drug (free SFB, NP-SFB, or NP-SFB-AB), with three wells per sample. The cells were incubated for an additional 72 hours. Next, 20 μL of MTT (Sigma, St. Louis, MO) in PBS (5 mg/mL) was added to each well, and the cells were incubated for an additional four hours at 37 °C. Next, 150 μL of DMSO were added to each well to dissolve the formed crystals, and the OD value of each well was measured at 490 nm. The number of viable cells correlated with absorbance, and indirectly reflected the number of viable cells. Cells treated with the same amount of PBS served as a control group.

### 2.7. Effect of SFB-loaded nanoparticles on the apoptosis of HepG2 cells

Six separate groups of HepG2 cells in their logarithmic growth phase were exposed to NP-SFB-Ab, NP-SFB, or SFB with an equivalent SFB concentration (5.0 μmol/L), or exposed to PBS alone, NPs without drug (null-NPs), or NP-Ab without drug (null-NPs-Ab) for 24 h, respectively. The cells were then enzymatically digested to create suspensions that were subsequently enriched by centrifugation. Next, approximately 490 μL of suspended cells was mixed with 5 μL of FITC-Annexin V plus 5 μL of PI (concentration 250 μg/mL) and incubated in an ice bath for 10 min. Following incubation, the cells were washed two times with PBS, and cellular apoptosis was detected with flow cytometry. Each analyzed sample contained 10,000 cells.

### 2.8. Kinase assay

Cell lysates were immunoprecipitated by gentle rotation overnight at 4 °C with anti-B-Raf or anti-C-Raf antibodies covalently coupled to Protein A/G Plus Sepharose beads. The immunoprecipitates were resuspended in 20 μL of a solution containing 0.5 mM β-glycerophosphate (pH 7.3), 1.5 mM ethylene glycol tetraacetic acid, 1 mM dithiothreitol, and 0.3% Brij35. Raf kinase activity was assessed by measuring phosphorylation of the kinase substrate (exogenous MEK). Briefly, a 20 μL aliquot of the kinase substrate, 2 μL of 1 mM ATP, and 2 μg of MEK-1 fusion protein (SignalChem, Richmond, Canada) were added to 20 μL of 50 mM MgCl_2_ solution and mixed with 20 μL of resuspended beads for 30 minutes. The reaction was stopped by the addition of sodium lauryl sulfate sample buffer. The reaction product was immunoblotted using anti-pMEK antibody and visualized with an enhanced chemiluminescence system.

### 2.9. Western blot assay

The expression levels of proteins in each experimental group were detected during the apoptosis experiments. Protein samples were subjected to SDS-PAGE, and the separated protein bands were transferred onto a nitrocellulose sheet, which was then blocked with skim milk at 4 °C overnight. The following morning, the membrane was incubated with diluted primary antibodies and a horseradish peroxidase-labeled secondary antibody for 30 min. The immunolabeled protein bands were stained with 3,3′-diaminobenzinine, and the nitrocellulose sheet was rinsed with water to stop the color staining reaction. β-Actin was used as an equivalent protein loading control.

### 2.10. Reactive oxygen species (ROS) assay

The ROS-sensitive fluorescent probe 2′,7′-dichlorodihydrofluorescein diacetate (H2DCFDA) is not fluorescent, but it can freely infiltrate into cells through their plasma membranes, and then is hydrolyzed to DCFH by intracellular esterases. However, DCFH cannot permeate the cellular membrane. ROS in cells oxidize non-fluorescent DCFH to form fluorescent DCF (excitation wavelength 488 nm, emission wavelength 525 nm). The cells in each experimental group were incubated with an appropriate volume of H2DCFDA (10 mmol/L) for 20 min in a 37 °C incubator. The cells were then washed with PBS, and their nuclei were stained with SYTO-17 for 15 min. The fluorescence intensity of DCF (H2DCFDA produced by enzymatic hydrolysis) was detected by fluorescence microscopy (Zeiss, Thornwood, NY) and used to determine the concentration of intracellular ROS. The experiment was repeated three times.

### 2.11. *In vivo* antitumor efficacy

A murine model of HCC was established and used to test the therapeutic efficacy of the prepared NPs *in vivo*. In brief, BALB/c nude mice (18–22 g), obtained from the Anhui Academy of Medical Science, were randomly assigned to six groups (PBS, Null-NPs, NP-Ab, SFB, NP-SFB, and NP-SFB-Ab; *n* = 15 mice per group). The mice in each group were subcutaneously injected into their right flank with 5 × 10^6^ HepG2 cells (150 μL/mouse) to establish the HepG2 xenograft model. The xenograft tumor spheres were allowed to reach a size of 50 mm^3^ before treatment was begun. The mice in each group received an intravenous injection of their treatment entity (PBS, Null-NPs, NP-Ab, SFB, NP-SFB, or NP-SFB-Ab; 5.0 μg/g/time on a SFB basis) into their tail vein on days 0, 4, 8, 12, and 16. The length and width of each tumor sphere were determined with a Vernier caliper, and tumor volume (*V*) was calculated as *V*=*d*^2^×*D*/2, where *d* and *D* are the largest and the smallest tumor diameter in mm, respectively. The body weight of each mouse was also measured as a means of evaluating systemic toxicity. After 4 weeks, the surviving mice in each group were sacrificed, and histopathologic changes in tissues and the immunostaining of target molecules were examined. The protocols for all animal experiments were approved by the Ethics Committee of the Anhui University of Science and Technology.

### 2.12. Statistical analysis

All experiments were performed at least three times, unless otherwise stated. The data were analyzed using SPSS Statistics for Windows, Version 17.0 (SPSS Inc., Chicago, IL), and differences between groups were analyzed using Student’s *t* test. Results are expressed as the mean ± SD. Differences with a *p* value <.05 were regarded as statistically significant.

## 3. Results and discussion

### 3.1. Characterization of polymers TPGS-b-PCL and pluronic P123-Mal

**Figure 2.**
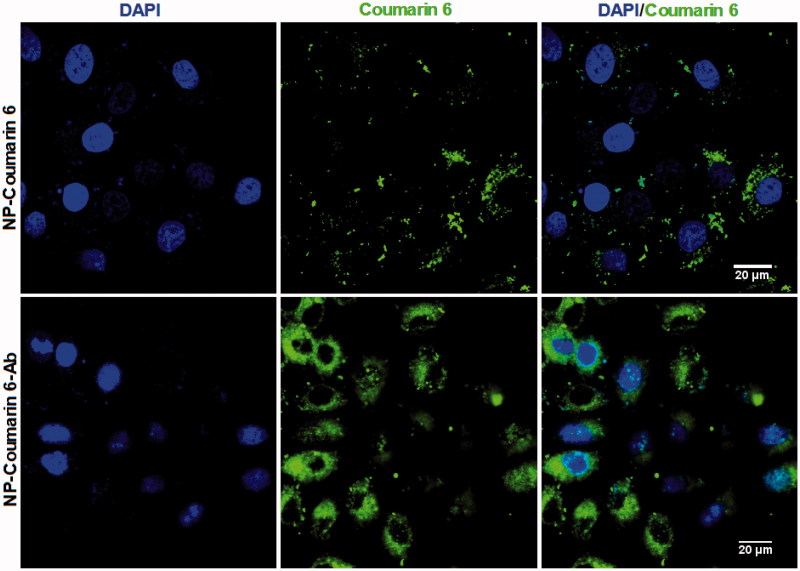
CLSM images of HepG2 cells after 4 h incubation with coumarin 6-loaded NP-Ab (NP-coumarin 6-Ab), and coumarin 6-loaded NP (NP-coumarin 6) at 37 °C. All samples have a coumarin-6 concentration of 2 μg/mL.

Fourier transform infrared (FTIR) analyses of TPGS-b-PCL/P123-Mal, the TPGS-b-PCL copolymer, and TPGS are presented in Figure 2(S). The wide and strong absorption band at 3486 cm^−1^ was due to the stretching vibration of water and the terminal hydroxyl group of the copolymer molecules. The absorption peak at 2978 cm^−1^ can be attributed to the methyl(–CH_2_) stretching vibration of PCL caused by stretching vibration of the terminal hydroxyl group. The absorption peak at 1732 cm^−1^ was due to the carbonyl vibration peak of TPGS. The absorption peak at 1107 cm^−1^ was caused by the C–O stretching vibration and vibration of the C–O–C skeleton in P123-Mal. The main absorption peaks described above in the copolymer prove that P123-Mal and TPGS-b-PCL were bound together.

### 3.2. Fabrication and characterization of the SFB-loaded polymeric nanoparticles

Newly obtained TPGS-*b*-PCL/P123-Mal copolymers were used to prepare SFB-loaded polymeric NPs (NP-SFB) that were then reacted with sulfhydryl-modified anti-GPC3 antibodies (Ab-SH) to give NP-SFB-Ab. The structure of the TPGS-*b*-PCL copolymer was validated by ^1^H NMR. As shown in Figure 1(A), there was a characteristic CDCl_3_ solvent peak at 7.28 ppm. The peaks at 4.08 ppm and 2.37 ppm were for –OCCH_2_– and –CH_2_OOC– on PCL, respectively; the single peak at 3.66 ppm was attributable to the methylene protons of TPGS. After calculating the ratio of integral areas between the PEG and PCL segments, the molecular weight of TPGS-*b*-PCL was determined as 1530 (Lin et al., 2017). The characteristic peaks of P123-Mal were those at 3.57, 3.42, and 1.15 ppm. The peak at 3.65 ppm was assigned to the -CH2 protons of the poly(ethylene oxide) portion of TPGS. Transmission electron microscopy showed that NP-SFB-Ab was uniformly distributed spherical particle with a size of approximately 115 nm (Figure 1(B)). The particle size of NP-SFB-Ab was larger than those of NP-SFB and Null-NPs, proving that its surface had been modified. These ^1^H NMR, TEM, and FTIR test results demonstrated that both PCL-b-TPGS and Pluronic P123-Mal polymers had been successfully prepared.

**Table 1.** Characterization of SFB-loaded polymeric nanoparticles.

NPs	*D*_h_ (nm)	PDI	ZP (mV)	LC (%)	EE (%)
Null-NP	95.3 ± 7.3	0.22	–12.8 ± 0.6		
NP-SFB	102.3 ± 6.3	0.15	–14.1 ± 0.5	10.1	77.1
NP-SFB-Ab	115.1 ± 8.2	0.18	–15.3 ± 0.8	9.9	75.9

*D*_h_: average hydrodynamic diameter; PDI: polydispersity index; ZP: zeta potential; NP: TPGS-b-PCL/P123-Mal nanoparticles; SFB: sorafenib; LC: drug loading content; EE: drug encapsulation efficiency.

As shown in Table 1, the size, size distribution, and zeta potential of NPs were measured by DLS. The mean particle size of NP-SFB-Ab was 115.1 nm, which was much smaller than those of NP-SFB (102.3 nm) and the Null-NP (95.3 nm). Moreover, the size distribution of NP-SFB-Ab (polydispersity index (PDI) = 0.18) was slightly narrower than that of the Null-NP (PDI = 0.22) and slightly larger than that of NP-SFB (PDI = 0.15). Zeta potential, which represents the surface charge of the NP, is a crucial criterion reflecting the stability of NP suspensions. As shown in Table 1, NP-SFB-Ab, NP-SFB, and the Null-NP had higher negative charges. The negative surface charge of NPs not only indicates the high stability of the NP suspension, but also the low toxicity of NPs to normal cells. As the cellular surface often carries a negative charge under normal circumstances, NPs do not easily penetrate cells, and therefore have sufficient dispersion stability. The values for LC and EE as detected by HPLC are also shown in Table 1. The drug LCs of NP-SFB and NP-SFB-Ab were 10.1% and 9.9%, respectively, and the drug encapsulation efficiencies of NP-SFB and NP-SFB-Ab were 77.1% and 75.9%, respectively. TPGS-b-PCL NPs demonstrated higher drug encapsulation efficiencies, which indicated efficient loading of SFB.

### 3.3. Stability and drug release profiles *in vitro*

**Figure 3.**
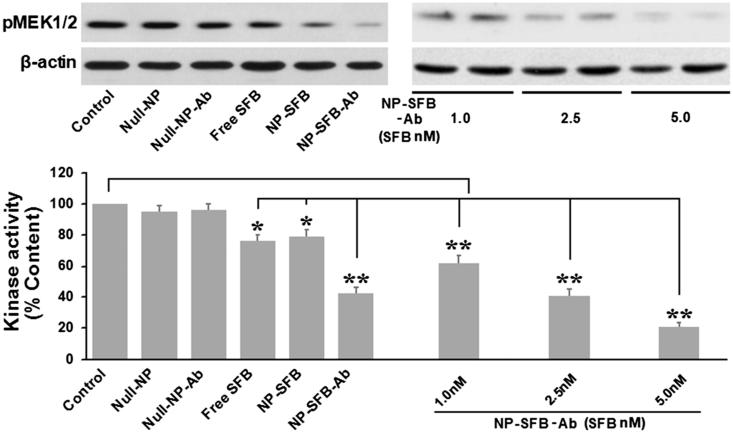
Sorafenib inhibited the kinase activity of c-Raf in HepG2 cells. Cells were treated for 12 h with different drugs (Control, Null-NP, Null-NP-Ab, Free SFB, NP-SFB, NP-SFB-Ab). c-Raf was immunoprecipitated and analyzed in vitro using MEK1/2 as a substrate. c-Raf kinase activity was assessed based on the optical density of phosphorylated MEK (pMEK) with respect to untreated cells. Free SFB, NP-SFB, and NP-SFB-Ab inhibited c-Raf kinase activity, while the c-Raf inhibited effect of NP-SFB-Ab was found with a significant decrease at doses from 1.0 nM (SFB content) and a significant inhibition at 5.0 nM (SFB content). *p < .05 versus control group; **p < .01 versus control group.

Our initial tests examined the release of SFB from NP-SFB-Ab and NP-SFB into cell medium during a 30-day period *in vitro*. SFB was released rapidly from NP-SFB within the first 14 days (>66.4%); however, the release rate decelerated during the subsequent 16 days. On day 30, 75.4% of the SFB had been released (Figure 3S). The rate of SFB release from NP-SFB-Ab was slightly faster than that from NP-SFB, probably because the NP surface became more hydrophilic after being modified by the Ab. As a result, the hydrophobic nuclei of NPs slightly swelled, and thereby promoted the release process. We next tested the *in vitro* stability of NP-SFB-Ab, as shown in Figure 3(S); the particle sizes of NP-SFB-Ab and NP-SFB in cell medium remained stable for 14 consecutive days.

### 3.4. Cellular uptake and localization of NPs

To study the uptake of NPs and NP-Ab by HepG2 cells, we encapsulated coumarin-6, a fluorescent agent, into NPs and NP-Ab, respectively. We then cultured the coumarin 6-loaded NP-Ab or coumarin 6-loaded NPs with HepG2 cells for 3 h, and observed their endocytosis by HepG2 cells with a CLSM (Olympus Fluoview FV-1000, Tokyo, Japan). The GPC3 protein is highly expressed on the surface of HepG2 cells (Dargel et al., 2015; Gao et al., 2015; Lin et al., 2017); therefore, NPs modified with anti-GPC3 antibody should be able to target those cells. As shown in Figure 2, cells cultured with coumarin 6-loaded NP-Abs displayed more intense fluorescence relative to cells cultured with coumarin 6-loaded NPs, indicating that more NPs were located in the cytoplasm, and thus could have a stronger effect on the cancer cells. Furthermore, the intracellular fluorescence of coumarin 6-loaded NP-Abs continued to increase over an extended time, and they targeted the HepG2 cells in a dose- and time-dependent manner. While the fluorescence of cells with coumarin 6-loaded NPs (coumarin 6-NPs) did not increase after reaching a certain intensity (experimental data is not shown), there was no evidence for the specific binding of coumarin 6-NPs in the cells. This finding suggests that the NP-Abs had specifically recognized and interacted with other Abs after the interaction between the Ab and GPC3, resulting in the active enrichment of Ab-NPs in the cell membrane. Subsequently, GPC3 molecules mediated intracellular endocytosis of NPs, thereby resulting in HepG2 cells being more efficiently enriched for Ab-modified NPs.

### 3.5. Biocompatibility assessment

**Figure 4.**
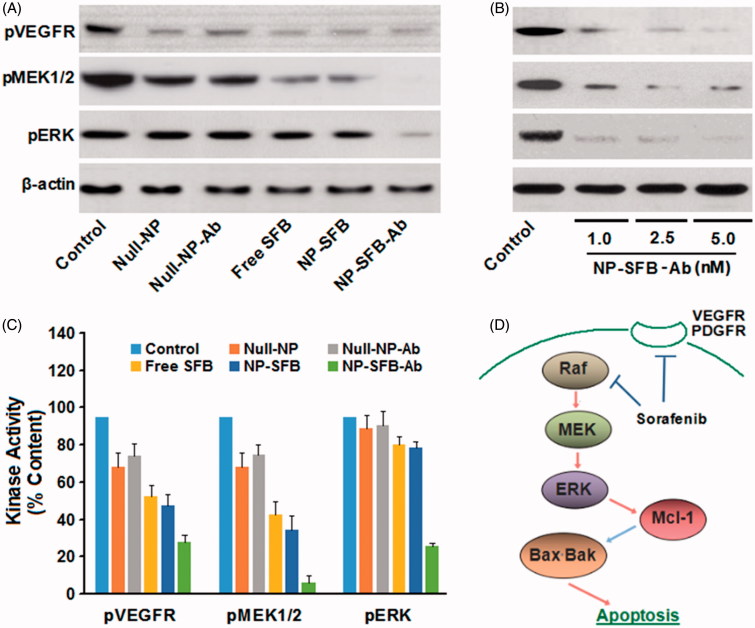
NP-SFB-Ab inhibits the phosphorylation of the MEK/ERK kinase signaling cascade and VEGFR in HepG2 cells.

To understand the compatibility of different SFB formulations with blood, the drug formulations were incubated with RBCs for 72 h, and the levels of hemolysis produced by exposure to free SFB and SFB-loaded NPs were measured. When the SFB concentration reached 1000 nM, which far exceeds the clinical treatment concentration, the rates of hemolysis produced by free SFB and SFB-loaded NPs (including NP-SFB and NP-SFB-Ab) were <5%, suggesting that the free SFB and SFB-loaded NPs were not toxic to RBCs. Next, the cytotoxicity of the SFB formulations was measured by the MTT assay (Figure 4S). After incubation with EAhy926 cells for 72 h, the NP-SFB-Ab formulation proved to be less toxic than free SFB or NP-SFB.

### 3.6. *In vitro* HepG2 cytotoxicity of SFB-loaded polymeric nanoparticles

**Figure 5.**
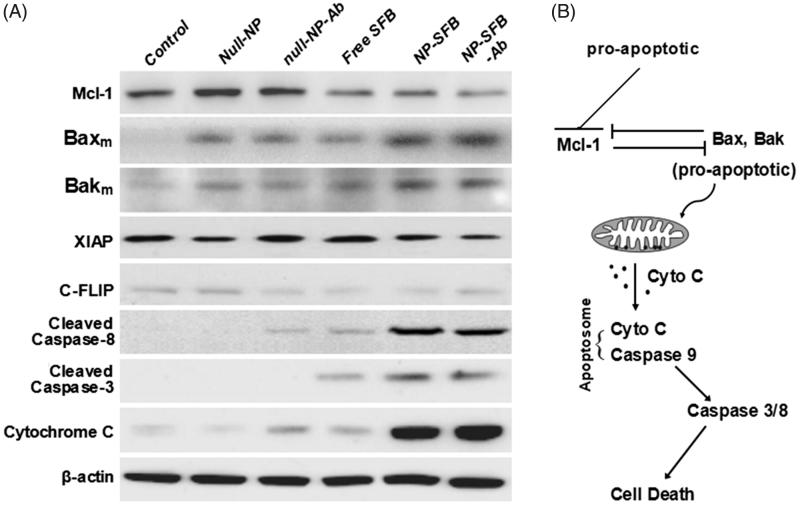
HepG2 cellular apoptosis induced by NP-SFB-Ab. In HepG2 cells treated with NP-SFB-Ab for 24 hours, the expression of antiapoptotic proteins XIAP and C-FLIP was not affected by western blot analysis, but Mcl-1 was inhibited to a certain extent; the levels of Bak and Bax on mitochondrial membrane were up-regulated; meanwhile, the expressions of apoptosis related proteins (hydrolyzed caspase-3, -8 and cytochrome c) were significantly up-regulated.

We used the MTT assay to test the ability of NP-SFB-Ab to kill HepG2 cells. Free SFB and NP-SFB with the same drug concentration, as well as non-drug-loaded NPs (Null-NPs), were used as controls. After 48 h of culture, NP-SFB-Ab exhibited the best killing effects (Figure 5S). Hence, modification with the Ab had significantly enhanced the cytotoxicity of SFB to HepG2 cells. This was consistent with results of our studies that examined SFB cellular uptake and SFB concentrations in cells. Moreover, that fact that NPs without SFB did not affect cellular growth supports the safety of the NPs.

### 3.7. Effect of NP-SFB-Ab on c-Raf kinase activity

As ERK phosphorylation is dependent on the upstream activation of Raf kinase, when HepG2 cells interact with drugs such as NP-SFB-Ab, NP-SFB, and SFB, the Raf kinase activity in HepG2 cells can be affected. The amount of substrate phosphorylated by exogenous MEK after immunoprecipitation of c-Raf from the HepG2 cellular lysates of various groups was used to indirectly detect c-Raf activity. The effect of SFB-loaded-NPs on c-Raf kinase activity *in vitro* was measured as shown in Figure 3. NP-SFB-Ab, NP-SFB, and free SFB all effectively inhibited c-Raf activity; however, NP-SFB-Ab was the most effective inhibitor, and inhibited c-Raf activity in a dose-dependent manner.

### 3.8. NP-SFB-Ab targets the downstream molecules RET and VEGFR, and blocks the MEK/ERK pathway by inhibiting phosphorylation of MEK and ERK

In HepG2 cells treated with NP-SFB-Ab, NP-SFB, or SFB, the intracellular levels of phosphorylated ERK were significantly reduced after 24 h (Figure 4), suggesting that like SFB, NP-SFB-Ab, and NP-SFB block the MEK/ERK pathway by suppressing Raf kinase activity. Therefore, in the HepG2 cell line, NP-SFB-Ab inhibits activation of the MEK/ERK pathway by targeting molecules such as Raf and VEGFR, and thereby it inhibits cell proliferation and promotes apoptosis.

### 3.9. NP-SFB-Ab induces apoptosis in HepG2 cells

To assess the mechanism of apoptosis in HepG2 cells induced with NP-SFB-Ab, HepG2 cells in the various groups were treated with equivalent 2.5 nM doses of SFB for 24 hours. Western blot analyses were then performed to assess the effect of those treatments on the expression levels of anti-apoptotic proteins XIAP, Mcl-1, and C-FLIP. The results showed that NP-SFB, SFB, and NP-SFB-Ab did not affect the level of antiapoptotic protein XIAP expression, but down-regulated the levels of anti-apoptotic proteins Mcl-1 and C-FLIP in HepG2 cells (Figure 5).

Mcl-1 plays a negative regulatory role in apoptosis through its binding to the Bcl-2 family of pro-apoptotic proteins (Bak, Bax, etc.) to form heterodimers. These heterodimers block the formation of Bax and Bak dimers on the mitochondrial membrane, and thereby inhibit the release of cytochrome C by mitochondria (Inoue-Yamauchi et al., 2017; Rashidi et al., 2018). Bik, NOXA, and tBid selectively bind to Mcl-1 when stimulated by apoptotic signals, resulting in the dissociation of Mcl-1 from Bak and Bax. Bak and Bax then form dimers on the mitochondrial membrane and enhance the permeability of the outer mitochondrial membrane to promote cytochrome C release, which leads to apoptosis (Brasacchio et al., 2017; Sivalingam et al., 2017). To clarify whether the down-regulation of Mcl-1 expression by NP-SFB-Ab affected the up-regulation of pro-apoptotic protein levels, we further examined how the various drugs affected Bax and Bak levels in the mitochondrial membrane. Our studies showed that the Bax and Bak levels in the mitochondrial membranes in the NP-SFB-Ab group were significantly higher than those in the control, SFB, and NP-SFB groups (Figure 5). High levels of Bax and Bak in the mitochondrial membrane would promote the polymerization of Bax and Bak, and thereby enhance the permeability of the mitochondrial outer membrane. This increase in permeability would promote the release of cytochrome C by mitochondria, and lead to apoptosis. Collectively, these results suggest that SFB induces caspase-dependent apoptosis and downregulates Mcl-1 and c-FLIP expression.

### 3.10. Effect of ROS production induced by NP-SFB-Ab on apoptosis

As ROS are involved in apoptosis (Ansari et al., 2017; Martínez-Torres et al., 2018), we further investigated whether ROS are involved in SFB-induced apoptosis. The molecule 2′,7′-dichlorodihydrofluorescein diacetate (DCFH-DA) can freely enter cells through the cell membrane and are hydrolyzed to DCFH by cellular esterases. DCFH cannot penetrate the cell membrane, and intracellular ROS can oxidize non-fluorescent DCFH to produce fluorescent DCF (Presti et al., 2017). Therefore, the detection of DCF fluorescence can be used to estimate the ROS levels in cells. Moreover, PARP, poly(ADP-ribose) polymerase plays an important role in DNA damage repair and is important for the stability and survival of cells. A loss of PARP enzyme activity will accelerate cellular instability. PARP can be cleaved by a variety of caspases *in vitro*, but it is mainly cleaved by caspase 3 *in vivo* (Zaker et al., 2017). The cleavage of human PARP between Asp124 and Gly215 by caspases causes PARP to lose its enzymatic activity by separating its catalytic domain (89 kDa) at the carboxy terminus from its amino-terminal DNA binding domain (24 kDa) (Zaker et al., 2017; Hsieh et al., 2018). Thus, PARP cleavage is an important indicator of apoptosis.

**Figure 6.**
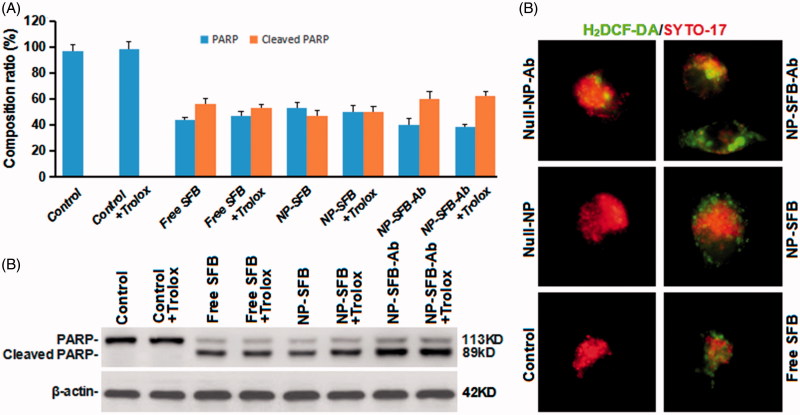
Effects of NP-SFB-Ab-induced ROS on apoptosis. PARP and cleaved PARP protein expression levels were detected by Western blotting after treatment with different drugs in combination with or without 100 μM trolox (reactive oxygen scavenger) for 24 hours. The values in (A) represent the mean ± SD from three independent samples. (B) Western blotting to detect PARP and cleaved PARP protein expression levels, β-actin levels were used as loading control. (C) The fluorescence intensity of H2DCF-DA of different group HepG2 cells treated with different drugs for 24 hours.

As shown in Figure 6, although NP-SFB-Ab induced higher levels of ROS production, a ROS scavenger (trolox) had no effect on apoptosis induced by NP-SFB-Ab treatment (Figure 6). Therefore, NP-SFB-Ab-induced apoptosis is probably not dependent on ROS signaling, and ROS signaling should not be considered the primary mechanism for SFB-induced apoptosis. Interestingly, Li et al. (Li et al., 2016) confirmed that SFB can inhibit AIB1 protein translation by inhibiting both the eIF4E translation initiation factor and the mTOR/p70S6K/RP-S6 signaling pathway, thereby reducing AIB1 expression in HCC cells. Downregulation of AIB1 protein expression can cause ROS in liver cancer cells to promote SFB-induced cellular death. Therefore, NP-SFB-Ab-induced ROS may have a synergistic effect on NP-SFB-Ab-induced apoptosis.

### 3.11. Antitumor efficacy *in vivo*

When the implanted HepG2 tumors reached 60 mm^3^ in size, the mice were injected with PBS, Null-NPs, NP-Ab, SFB, NP-SFB, or NP-SFB-Ab. As shown in Figure 6(S), the mean tumor volumes of mice treated with PBS, Null-NP, NP-Ab, SFB, or NP-SFB continued to increase to 357, 330, 339, 221, and 163 mm^3^, respectively, at 44 days after the first drug injection, whereas the mean tumor volume in the NP-SFB-Ab group increased to only 150 mm^3^. Therefore, NP-SFB-Ab inhibited tumor growth more effectively than did the other agents.

## 4. Conclusions

A novel SFB-loaded nanoparticle (NP-SFB-Ab) was successfully developed for use in the targeted therapy of liver cancer. NP-SFB-Ab had good stability and released the anticancer drug SFB. NP-SFB-Ab demonstrated an ability to selectively target GPC3 positive cells and displayed higher levels of cellular uptake than did non-targeted NP-SFB and free SFB. NP-SFB-Ab also had a promising toxicological profile and was more cytotoxic to HCC cells than were non-targeted NP-SFB and free SFB. *In vivo* studies confirmed that NP-SFB-Ab inhibited tumor growth to a greater extent than did NP-SFB or free SFB. Our results confirmed that NP-SFB-Ab downregulates MEK 1/2 and ERK phosphorylation by inhibiting Raf kinase activity, and it also inhibits the RAF/MEK/ERK signaling pathway. Moreover, NP-SFB-Ab also downregulated Mcl-1 expression, resulting in polymerization of Bax and Bak on the mitochondrial membrane. This polymerization enhances mitochondrial permeability, promotes the release mitochondrial cytochrome C, and thereby induces apoptosis. As a result, this polymer NP should be considered for the targeted treatment of liver cancer.

## Supplementary Material

Supplemental_Materials.pdf
