# Supplementary material for: Anti-GPC3 antibody-modified sorafenib-loaded nanoparticles significantly inhibited HepG2 hepatocellular carcinoma
Source: Drug Deliv. 2018 Jun 19;25(1):1484–94. doi: 10.1080/10717544.2018.1477859 (PMC6058710; doi:10.1080/10717544.2018.1477859)
Supplement: Supplemental_Materials.pdf [file IDRD_A_1477859_SM6330.pdf]

Table 1S. Characterization of SFB-loaded polymeric nanoparticles[a].

NPs	D_h (nm)	PDI	ZP (mV)	LC (%)	EE (%)
Null-NP	95.3 ± 7.3	0.22	-12.8 ± 0.6		
NP-SFB	102.3 ± 6.3	0.15	-14.1 ± 0.5	10.1	77.1
NP-SFB-Ab	115.1 ±8.2	0.18	-15.3 ± 0.8	9.9	75.9

Note: D_h, average hydrodynamic diameter; PDI, polydispersity index; ZP, zeta potential; NP, TPGS-b-PCL/P123-Mal nanoparticles; SFB, Sorafenib; LC, drug loading content; EE, drug encapsulation efficiency.

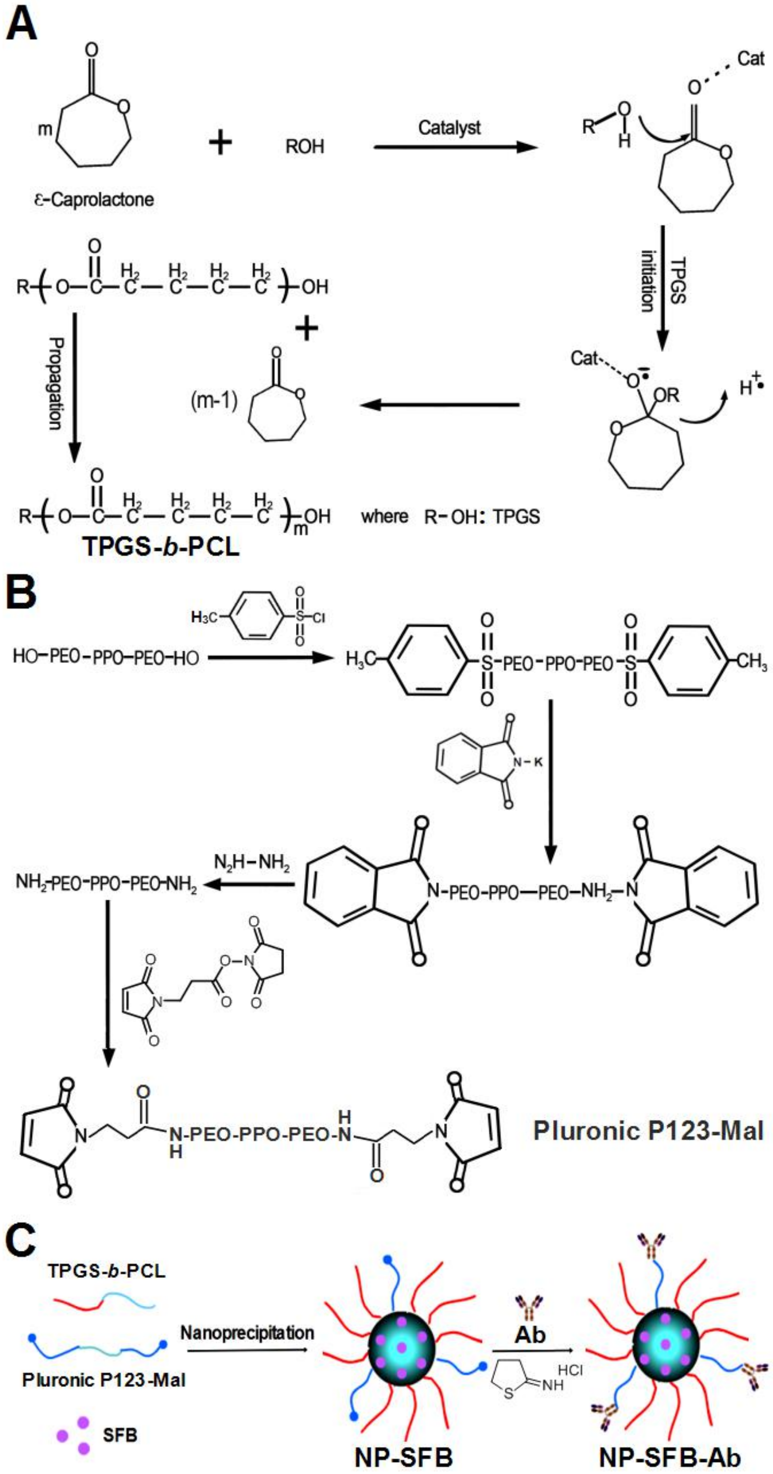

Fig. 1S Synthesis of TPGS-b-PCL (A), Pluronic P123-Mal (B) and NP-SFB-Ab(C).

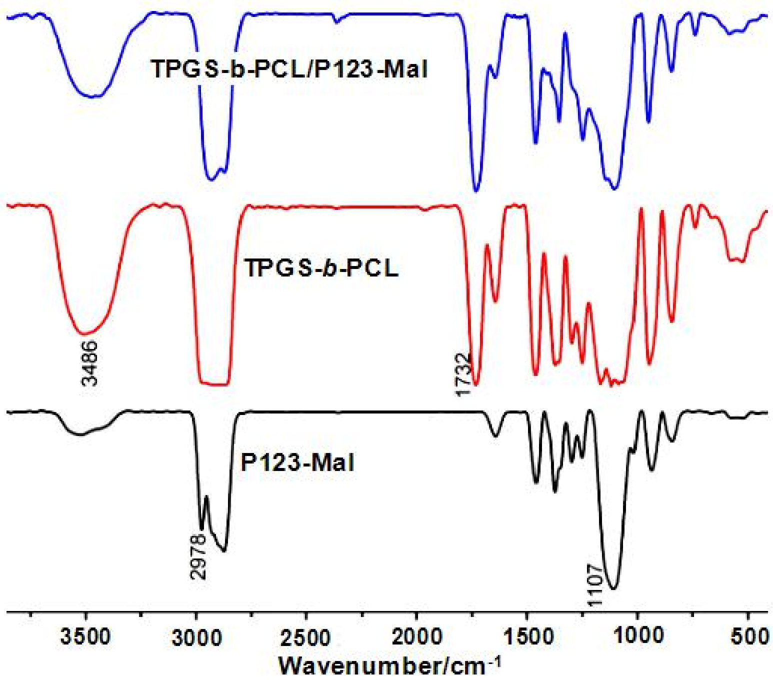

Figure 2S FTIR spectra of P123-Mal, TPGS-b-PCL and TPGS-b-PCL/P123-Mal copolymer.

Abbreviations: FTIR, Fourier transform infrared; PCL, poly(ε-caprolactone); TPGS, D-α-tocopheryl polyethylene glycol 1000 succinate.

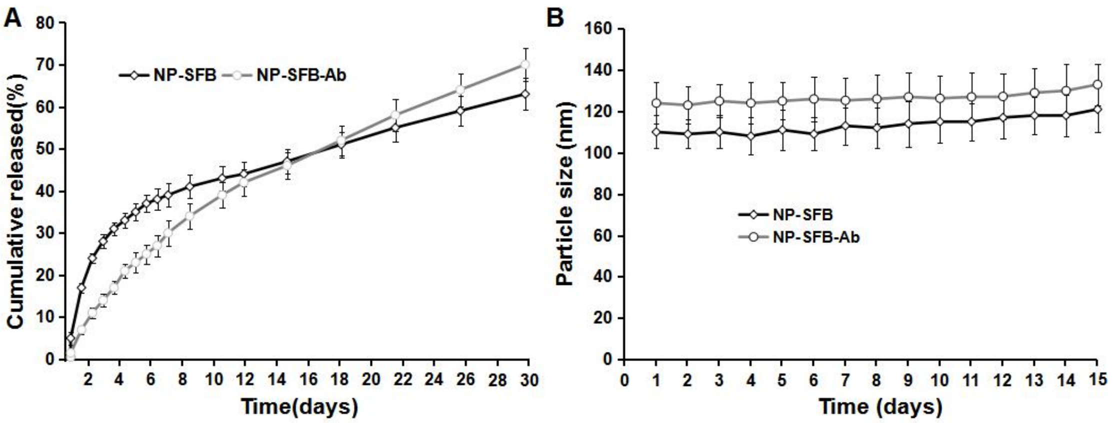

Figure 3S. Stability and drug cumulative release efficiency of drug-loaded nanoparticles. (A) Cumulative SFB release of NP-SFB and NP-SFB-Ab in cell medium over 30 days; (B) Size changes of NP-SFB and NP-SFB-Ab incubated in cell medium containing 10% FBS over 14 d.

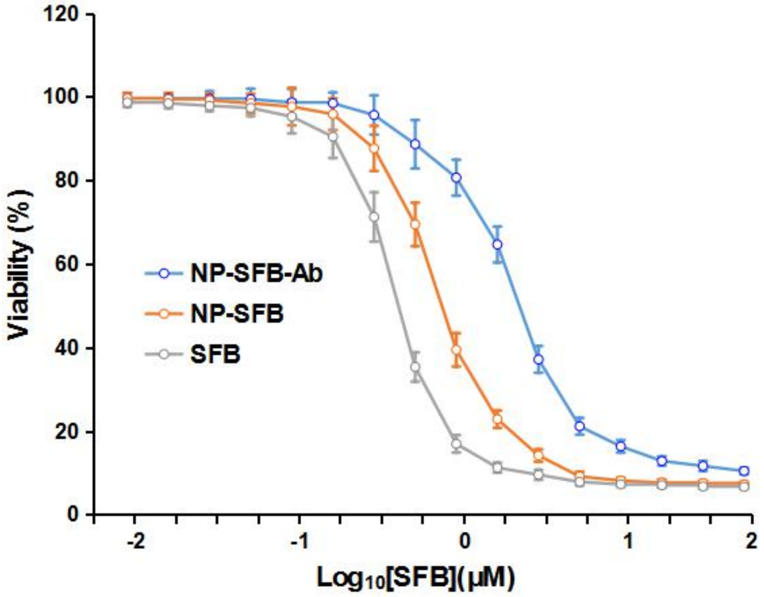

Figure 4S In vitro cytotoxicities of SFB-loaded formulations. Cytotoxicity of free SFB and SFB-loaded formulations against Eahy926 cells with the MTT assay. Data from three independent experiments were expressed as mean ± SEM (n=3).

Abbreviations: MTT, 3-(4,5-dimethylthiazol-2-yl)-2,5-diphenyltetrazolium bromide; SFB, Sorafenib.

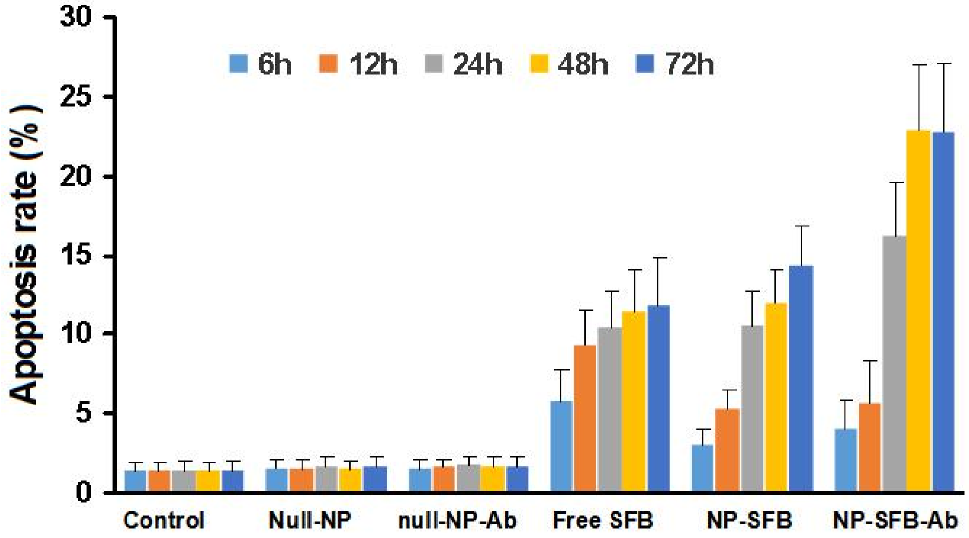

Figure 5S Cell apoptosis rate of HepG2 cells treated by different drug.

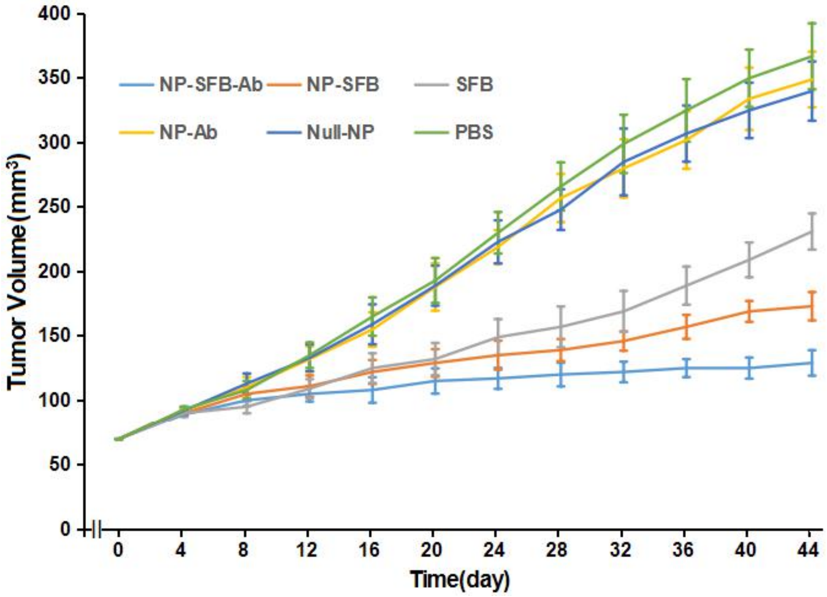

Figure.6S Antitumor efficacy of NP-SFB-Ab upon HepG2 xenograft-bearing nude mice.
